# The Role of General Practice in Complex Health Care Systems

**DOI:** 10.3389/fmed.2021.680695

**Published:** 2021-11-25

**Authors:** Katharina Schmalstieg-Bahr, Uwe Wolfgang Popert, Martin Scherer

**Affiliations:** ^1^Department of General Practice and Primary Care, University Medical Center Eppendorf, Hamburg, Germany; ^2^Department of General Practice, University Medical Center Göttingen, Göttingen, Germany

**Keywords:** general practice, health care system, gatekeeping, continuity of care, comprehensive care, core values

## Abstract

According to the WHO, in a complex system, “there are so many interacting parts that it is difficult (…), to predict the behavior of the system based on knowledge of its component parts. “In countries without general practitioner (GP)-gatekeeping, the number of possible interactions and therefore the complexity increases. Patients may consult any doctor without contacting their GP. Family medicine core values, e.g., comprehensive care, and core tasks, e.g., care coordination, might be harder to implement and maintain. How are GPs perceived and how do they perceive themselves if no GP-gatekeeping exists? Does the absence of any GP-gatekeeping influence family medicine core values? A PubMed and Cochrane search was performed. The results are summarized in form of a narrative review. Four perspectives regarding the GP's role were identified. The GPs' self-perception regarding family medicine core values and tasks is independent of their function as gatekeepers, but they appreciate this role. Patient satisfaction is also independent of the health care system. Depending on the acquisition of income, specialists have different opinions of GP-gatekeeping. Policymakers want GPs to play a central role within the health care system, but do not commit to full gatekeeping. The GPs and policymakers emphasize the importance of family medicine specialty training. Further international studies are needed to determine if family medicine core values and tasks can be better accomplished by GP-gatekeeping. Specialty training should be mandatory in all countries to enable GPs to fulfill these values and tasks and to act as coordinators and/or gatekeepers.

## Introduction

A complex system can be defined in various ways (1–4). According to the WHO, it is a system in which “there are so many interacting parts that it is difficult, if not impossible, to predict the behavior of the system based on knowledge of its component parts.” Delivering health care in general meets this definition due to, for example, the huge number of relationships between patients, caregivers, health care providers, support staff, family, and community members, the diversity of tasks as well as the diversity of care pathways, and organizations involved (5). In countries in which general practitioners (GP)/family physicians do not function as gatekeepers to the health care system, e.g., in Austria, the Czech Republic, and Greece (6), the number of possible interactions and therefore the complexity increases. Patients are not required to have a GP and/or may consult any doctor of any specialty without contacting their GP first. This could be resource-intensive, as it may lead to unnecessary patient-doctor encounters, or potentially be harmful if diagnostic tests are doubled, not ordered at all, or drug interactions occur due to a lack of coordination.

Family medicine principles have been described and redefined over the years (7–9). In 2019, van der Horst and Wit identified four core values through a complex discussion and voting process involving more than 1,000 GPs: continuity, medical generalism, person-centeredness, and collaboration (with patients, colleagues, and other health care professionals). Furthermore, they specified medical generalist care, out-of-hour services, palliative- and preventive care as well as coordination of care as core tasks (7, 10). Especially, values, such as continuity and medical generalism/comprehensiveness or tasks, such as coordination of care might be harder to implement and maintain in complex medical systems without any gatekeeping.

This article focuses on the challenges for GPs in complex health care systems. How do GPs perceive their own role if no GP-gatekeeping exists? How do others perceive the role of the GP? Does the absence of any GP-gatekeeping influence family medicine core values?

## Methods

### List of Countries

Based the report of the Organisation for Economic Co-operation and Development (OECD) and European Union (EU) (6), the following countries were identified as countries without GP-gatekeeping (Table 1).

**Table 1:** Countries without GP-gatekeeping.

	**No GP gatekeeping**	**Financial incentives to obtain a referral, but direct access is possible**
**EU-countries**		
Austria	X	
Belgium		X
Cyprus	X	
Czech Republic	X	
Denmark		X
Estonia^a^	(X)	
France		X
Germany	X	
Greece	X	
Latvia		X
Luxembourg	X	
Malta		X
Romania		X
Slovak Republic		X
United Kingdom^b^	(X)	
**Non-EU countries**		
Iceland		
Switzerland		X
Turkey	X	

a *GP gatekeeping, but direct access to a dermatologist, ophthalmologist, gynecologist, and psychiatrist is possible*.

b *Primary-care physician referral is the usual way of accessing secondary care, but patients can also refer themselves for secondary care without consulting a GP*.

### Literature Review

A narrative, rather than a systematic, review was chosen to give an overview, and cover a wide range of issues within the topic at the same time. Although narrative reviews often do not reveal explicit information about the literature search and why studies were found to be relevant (11), the authors of this article decided to include this information to increase transparency.

A PubMed and Cochrane search was performed. In PubMed, Medical Subject Headings (MeSH) were used to identify relevant literature regarding the role of GPs in health care systems without gatekeeping. Since the MeSH-terms care continuity and patient-centered care were indexed under comprehensive health care, the third and fourth search showed fewer results. The second to fourth search did not yield any additional relevant articles that had not been included before. In Cochrane, an advanced search was performed. The filter was set to “all text” and the same headings as in PubMed were used (Table 2). All full-text articles in English and German that met the topic were included. No filter regarding the publication date was applied.

**Table 2:** Search strategy.

	**Results**	**Relevant**	**Newly included**
**PubMed medical subject headings**			
(Family Medicine[MeSH Terms]) AND (gatekeeping[MeSH Terms])	81	15	15
((Family Medicine[MeSH Terms]) AND (gatekeeping[MeSH Terms])) AND (comprehensive health care[MeSH Terms])	28	7	0
((Family Medicine[MeSH Terms]) AND (gatekeeping[MeSH Terms])) AND (care continuity, patient[MeSH Terms])	4	1	0
((Family Medicine[MeSH Terms]) AND (gatekeeping[MeSH Terms])) AND (patient centered care[MeSH Terms])	3	1	0
**Cochrane headings**			
“family medicine” in All Text AND “gatekeeping” in All Text	8	0	0
“family medicine” in All Text AND “gatekeeping” in All Text AND comprehensive health care in All Text	4	0	0
“family medicine” in All Text AND “gatekeeping” in All Text AND care continuity in All Text	7	0	0
“family medicine” in All Text AND “gatekeeping” in All Text AND patient centered care in All Text	7	0	0

## Results

Fifteen relevant articles from 2000 to 2017 were found by the PubMed search. The list is shown under Supplementary Table 1. Further relevant literature was identified while reviewing the articles (12–24).

### The GP's Perspective

The GP's self-perceived responsibilities and aspirations regarding family medicine core values and core tasks do not seem to depend on the health care system he or she practices in. Sturm described continuity of care while using the knowledge about the patient's social situation as essential and called for ongoing responsibility during and after the consultation and while receiving secondary care, although German GPs do not generally function as gatekeepers (25). In Israel, patients may consult any GP or specialist within their health care plan, although the country has tried to move toward a gatekeeping system (26, 27). Nonetheless, the study of Tabenkin et al. showed that almost all participating GPs considered “coordination of all patient care” as very important, a third-rated “24 h responsibility for patients” as important (15). But the traditional role of the GP as the first contact person within the health care system may shift from the individual to the practice, where the GP leads a team that collectively takes responsibility and provides a patient-centered medical home (28).

Several studies demonstrated that GPs prefer the gatekeeper role (24, 29–31) due to multiple reasons, e.g., GPs in Iceland thought that mandatory referral increased the flow of information and enhanced the communication with a specialist (32). Fewer hospital admissions, better quality of care, and lower health care cost were also mentioned (24). Rosemann et al. showed that not only the patient had a better experience with a referral, but also the GP if he or she was the initiator of the referral (30). But there are also critical voices among primary-care scientists (12). Greenfield et al. called for a revision of gatekeeping regulations in the United Kingdom, where GP-gatekeepers are established, to grant patients more choices and by that “facilitate more collaborative work” with other specialties (23). A Lithuanian study suggested a flexible gatekeeping model regarding adolescents' reproductive health care, as GPs questioned the appropriateness of gatekeeping in this field due to a lack of willingness to provide these services, insufficient training, and inadequately equipped surgeries (33). GPs considered formal specialty training as essential (15).

The GP's choice of specialists may depend on the health care system, as they are sometimes required to refer patients within a network (34).

### The Patient's Perspective

A large study that included 17,391 patients in 10 different countries (with and without gatekeeping) evaluated the patient's view on general practice and found no large discrepancies in regards to aspects of care. Minor differences were the relatively positive evaluations given to preventive services in the United Kingdom and the GP's availability (either by an appointment or by phone) in Switzerland, Germany, and Belgium. The relatively negative evaluations were given for service in case of emergencies in the United Kingdom and Slovenia. Slovenian patients also gave relatively negative evaluations regarding the GP's interest in their personal situation. A tendency toward a more positive overall assessment was seen in Switzerland, Germany, and Belgium (countries with no GP-gatekeeping). For the United Kingdom and the Scandinavian countries, a trend toward a less positive assessment was found (22).

In Germany, multiple attempts have been made to shift to a more family medicine-centered care model. Although it has only been established in voluntary projects, Himmel et al. showed that the majority of over 400 participants from the general population would accept their GP as gatekeeper and appreciated the coordination of secondary care by the GP. Nearly two-thirds wanted to consult their GP during hospitalization. Participants who had a GP at the time of the survey were more likely to accept him or her as gatekeeper compared with participants without a GP (35). The results are in line with a study from the United States (17). Another study from Israel reported numbers that were less clear, but trending toward the same direction: a third of all respondents preferred self-referral to a specialist, 40% preferred their GP to act as a gatekeeper, and 19% preferred the GP to coordinate care but to refer themselves to a specialist (13, 15). A few years before, 52% of the respondents were in favor of direct access to specialists, but the rate was lower in patients who were older than 45 years and patients whose primary-care physician was a specialist in family medicine (16). The denial of a referral resulted in lower satisfaction rates (20). On the contrary, a patient's experience was more positive if the initiative for a referral came from the GP. The authors concluded that this supports the GP's role as gatekeeper, since, in Germany, the patients could have directly scheduled an appointment with a specialist (30). However, satisfaction rates were not compared to patients who had opted to do so.

Although gatekeeping is often focused on when accessing the patient's perspective on the GP role, van den Brink-Muinen et al. demonstrated that the doctor-patient communication was hardly influenced by it. They compared data from countries with and without gatekeeping and only found that paraphrases, checks for understanding, and requests for clarification and opinion were found more often in consultations of the gatekeeping countries (36). Two newer reviews conclude that evidence regarding the effect of gatekeeping on quality of care and patient or provider satisfaction is inconsistent and limited (18, 19). A large international study using survey data from over 25,000 patients in 17 countries showed that patients were highly satisfied with their GPs, independent of health care system characteristics such as GP density, fee for service reimbursement, gatekeeping, or the GP's role as first contact (37).

### The Specialist's Perspective

Among a U.S. group of nearly 1,500 specialists (cardiology, endocrinology, gastroenterology, general surgery, neurology, ophthalmology, and orthopedics), the attitudes toward primary-care gatekeepers were mixed. Compared with non-salaried physicians, salaried physicians were more in favor of gatekeepers, as did physicians with a greater percentage of practice income derived from capitation (21). A study from The Netherlands showed that specialists were particularly interested in collaborating with GPs due to their function as gatekeepers. However, an informal network with incidental contacts fulfilled the collaborative needs of the specialists. They did not regard GPs as equal and felt that GPs could learn a lot from them, but that there was nothing to learn *vice versa* (38). Specialists were satisfied with the appropriateness and timing of the referral but would have appreciated more information about the patient's medical history or medications (30). If multiple specialties or even professions address the same medical problem, it is likely that more than one will claim the gatekeeper role. A study among ophthalmologists, GPs, orthoptists, optometrists, and opticians regarding common eye problems was at least able to show a trend toward a medical (ophthalmologist, GP) rather than a non-medical gatekeeper (optometrist) (31).

### The Policymaker's Perspective

Compared with the other three groups, the policy maker's perspective regarding the GP's role in complex medical systems is less well-researched. Mariñoso and Jelovac used a statistical model to identify optimal contracts that would induce the best behavior from a public insurer's point of view and found that gatekeeping was superior wherever GP's incentives matter (39). Philips et al. performed a multivariate logistic regression analysis using secondary data showing that gatekeeper requirements are associated with higher utilization of widely recommended cancer screening interventions (e.g., mammography). No association was found regarding the use of less uniformly recommended interventions [e.g. prostate-specific antigen (PSA) checks]. The authors concluded that “policymakers should consider the potential benefits of gatekeeper requirements with respect to preventive care when designing health plans and legislation” (40).

Only two studies from Israel reported data directly displaying the policymaker's perspective (14, 15). The members of the Ministry of Health, the Sick Funds' central administrations, and the Israel Medical Association (IMA) central office were interviewed and stated that the highly trained GP should play a central role in the health care system. GPs should be highly accessible coordinators, able to weigh cost considerations. However, only about half of the participants supported a GP-gatekeeper model. The perceived barriers to implement such a model included loss of faith in GPs by the general population, dearth of GPs with adequate training, low stature, lack of 24 h-availability, resistance from specialists, and competition between the sick funds.

## Discussions

Most publications illustrate the GP's and patient's point of view. Lesser is known about the view of secondary-care providers, especially addressing other aspects of the GP's role, besides his or her function as a gatekeeper. Studies depicting the policymaker's perspective are lacking. Few studies directly compare countries with and without GP-gatekeeping. Often studies focus on the question whether GP-gatekeeping should be implemented, is beneficial, or leads to satisfaction of patients, GPs, and specialists. Other aspects of the GP's role in complex health care systems are lesser well-researched.

The GP's self-perceived role regarding family medicine core values and core tasks is independent of the health care system. The GPs strive to accomplish these whether they function as gatekeepers or not, but there is evidence that they appreciate or would appreciate this role. Patient satisfaction and doctor-patient communication also seem to be independent of the health care system. Depending on the acquisition of income (salaried vs. non-salaried physicians, capitation vs. no capitation), specialists have different opinions on whether GPs should function as gatekeepers, but do not regard them as equals. Policymakers want GPs to play a central role within the health care system, but do not like to commit to full GP-gatekeeping. The GPs and policymakers emphasize the importance of family medicine specialty training.

Besides the health care system, cultural, religious, economical, and even geographical aspects might influence the role of the GP and implementation of family medicine core values.

### Limitations

As this is a narrative review, the typical limitations apply: compared to a systematic review, the literature search was more subjective and less structured. The aim was to give an overview of the GP's role in a complex medical system without gatekeeping. It is possible that a more systematic research and the use of other/further MeSH terms would have identified more articles, although relevant literature was added while reviewing the initial list. The aim of the review is to describe the GP's role in health care systems that are similar to each other (no GP-gatekeeping) without focusing on a national perspective. All authors have worked and lived in other countries. However, it is possible that the article was influenced by their experience in Germany. Despite the fact that the debate of the GP's role is ongoing, most articles are over 10 years old.

## Conclusions

Further international studies are needed to compare the role of GPs in countries with and without gatekeeping. These studies should include multiple perspectives (GP, patient, specialist, and policymaker) and go beyond the question of whether GP-gatekeeping should be established or not. There are studies indicating that specialty training needs to be mandatory in all countries to enable GPs to fulfill family medicine core values and tasks and to act as coordinators and/or gatekeepers. However, more studies are needed to prove this.

## Author Contributions

KS-B conducted the literature review. All authors were responsible for drafting the manuscript and for the critical revision of the content.

## Conflict of Interest

The authors declare that the research was conducted in the absence of any commercial or financial relationships that could be construed as a potential conflict of interest.

## Publisher's Note

All claims expressed in this article are solely those of the authors and do not necessarily represent those of their affiliated organizations, or those of the publisher, the editors and the reviewers. Any product that may be evaluated in this article, or claim that may be made by its manufacturer, is not guaranteed or endorsed by the publisher.
