# Supplementary material for: The Role of General Practice in Complex Health Care Systems
Source: Front Med (Lausanne). 2021 Nov 25;8:680695. doi: 10.3389/fmed.2021.680695 (PMC8655229; doi:10.3389/fmed.2021.680695)
Supplement: Supplementary file 1 [file Table_1.docx]

**Table 1: Overview of incluced publications**

| Puplication | Country | Type of study | Perspective |
| --- | --- | --- | --- |
| Tabenkin H, Gross R. The role of the primary care physician in the Israeli health care system as a 'gatekeeper'--the viewpoint of health care policy makers. Health Policy. 2000 Jun;52(2):73-85. | Israel | Structured interviews | Policy makers |
| Himmel W, Dieterich A, Kochen MM. Will German patients accept their family physician as a gatekeeper? J Gen Intern Med. 2000 Jul;15(7):496-502. | Germany | Cross-sectional telephone survey among the general population | Patients / general population |
| Fairchild DG, Sussman AJ, Lee TH, Brennan TA. When sick patients switch primary care physicians: the impact on AMCs participating in capitation. Acad Med. 2000 Oct;75(10):980-5. | USA | Expert comment | - |
| Stevens FC, van der Horst F, Hendrikse F. The gatekeeper in vision care. An analysis of the co-ordination of professional services in The Netherlands. Health Policy. 2002 Jun;60(3):285-97 | Netherlands | Questionnaire survey | GPs / specialists |
| Mariñoso BG, Jelovac I. GPs' payment contracts and their referral practice. J Health Econ. 2003 Jul;22(4):617-35. | UK | Statistic model to identify the optimal contracts | (Public insurer) |
| van den Brink-Muinen A, Verhaak PF, Bensing JM, Bahrs O, Deveugele M, Gask L, Mead N, Leiva-Fernandez F, Perez A, Messerli V, Oppizzi L, Peltenburg M. Communication in general practice: differences between European countries. Fam Pract. 2003 Aug;20(4):478-85. | Netherlands, Spain, UK, Belgium, Germany, Switzerland | Patient and GP questionnaires and observation of videotaped consultations | Patients / GPs |
| Rice B. If you're losing patients to specialists... Med Econ. 2003 Sep 18;80(19):27-8, 31. | USA | Expert comment | GPs |
| Wensing M, Baker R, Szecsenyi J, Grol R; EUROPEP Group. Impact of national health care systems on patient evaluations of general practice in Europe. Health Policy. 2004 Jun;68(3):353-7.. | 17 countries | Patient survey data and data-bases for health care system characteristics | Patients |
| Phillips KA, Haas JS, Liang SY, Baker LC, Tye S, Kerlikowske K, Sakowski J, Spetz J. Are gatekeeper requirements associated with cancer screening utilization? Health Serv Res. 2004 Feb;39(1):153-78. | USA | Cross-sectional, multivariate logistic regression analysis using secondary data | - |
| Sturm D. Hausarztbasierte Versorgung [Family doctor-based health care]. Z Kardiol. 2005;94 Suppl 4:IV/1-3. German. | Germany | Expert comment | GPs |
| Rosemann T, Wensing M, Rueter G, Szecsenyi J. Referrals from general practice to consultants in Germany: if the GP is the initiator, patients' experiences are more positive. BMC Health Serv Res. 2006 Jan 19;6:5. | Germany | Questionnaire surveys | GPs, specialists and patients |
| Jaruseviciene L, Levasseur G. The appropriateness of gatekeeping in the provision of reproductive health care for adolescents in Lithuania: the general practice perspective. BMC Fam Pract. 2006 Mar 14;7:16. | Lithuania | Interviews | GPs |
| Berendsen AJ, Benneker WH, Schuling J, Rijkers-Koorn N, Slaets JP, Meyboom-de Jong B. Collaboration with general practitioners: preferences of medical specialists--a qualitative study. BMC Health Serv Res. 2006 Dec 4;6:155. | Netherlands | Semi-structured interviews | Specialists |
| Bjornsson S, Sigurdsson JA, Svavarsdottir AE, Gudmundsson GH. Gatekeeping and referrals to cardiologists: general practitioners' views on interactive communications. Scand J Prim Health Care. 2013 Jun;31(2):79-82. | Iceland | Cross-sectional questionnaire survey | GPs |
| Lemire F. First contact: what does it mean for family practice in 2017? Can Fam Physician. 2017 Mar;63(3):256. | Canada | Expert comment | GPs |
